# Both reduced ovarian reserve and severe semen alterations are overrepresented in couples seeking assisted reproductive technology treatment for the first time: a cross-sectional study

**DOI:** 10.1186/s12978-023-01666-0

**Published:** 2023-08-16

**Authors:** Juan J. Tarín, Eva Pascual, Miguel A. García-Pérez, Aitana Monllor-Tormos, Antonio Cano

**Affiliations:** 1 Department of Cellular Biology, Functional Biology and Physical Anthropology, Faculty of Biological Sciences, University of Valencia, Burjassot, Valencia, Spain; 2 Institute of Health Research INCLIVA, Valencia, Spain; 3 Department of Genetics, Faculty of Biological Sciences, University of Valencia, Burjassot, Valencia, Spain; 4 Service of Obstetrics and Gynecology, University Clinic Hospital, Valencia, Spain; 5 Department of Pediatrics, Obstetrics and Gynecology, Faculty of Medicine, University of Valencia, Valencia, Spain

**Keywords:** Female infertility, Infertility diagnosis, Infertility duration, Male infertility

## Abstract

**Background:**

Once a mate choice decision has been made, couples that fail to reach a live birth in natural and/or intrauterine insemination (IUI) cycles will likely visit fertility clinics seeking assisted reproductive technology (ART) treatment. During the more or less prolonged period of infertility experienced, those couples with mild/moderate reproductive anomalies would have advantage over couples displaying more severe reproductive alterations in achieving a natural or IUI conception. Thus, we can expect to find a progressive increase in the proportion of couples with more severe reproductive anomalies as duration of infertility rises. In this study, we aim to ascertain whether there is an association between male and female infertility diagnoses and duration of infertility in couples seeking ART treatment for the first time.

**Methods:**

A cross-sectional analysis of 1383 infertile couples that sought ART treatment for the first time. Forward-stepwise binary logistic regression analyses were applied to calculate exponentiated regression coefficients.

**Results:**

Men suffering from any combination of oligo-, astheno-, and teratozoospermia (ACOAT) exhibited higher odds of having a duration of infertility > 2 years compared with non-ACOAT men [odds ratio (95% confidence interval): 1.340 (1.030–1.744)]. Women from ACOAT couples displaying a duration of infertility > 2 years presented shorter menstrual cycles (P ≤ 0.047) and lower antral follicular count (AFC) values (P ≤ 0.008) and serum anti-Müllerian hormone (AMH) levels (P ≤ 0.007) than women from non-ACOAT couples exhibiting > 2 years of infertility. Likewise, AFC values (P ≤ 0.013) and serum AMH levels (P ≤ 0.001) were decreased when compared with women from ACOAT couples displaying ≤ 2 years of infertility. A relative low but significant percentage of ACOAT couples displaying > 2 years of infertility stood out for their smoking habits.

**Conclusions:**

Couples consisting of ACOAT men and women with a relative low ovarian reserve are overrepresented in couples seeking ART treatment for the first time after experiencing > 2 years of infertility. This outcome leads us to develop a general hypothesis proposing that the origin of couple’s infertility is a consequence of a process of positive assortative mating shaped by sexual selection forces.

## Introduction

Each person has his/her own mating preferences. These include social, behavioral or personality characteristics such as education level, occupation, socioeconomic status, smoking, alcohol consumption, language, culture, kindness, and honesty; as well as physical traits such as age, height, skin pigmentation, eye and hair color, and physical attractiveness that provide cues of resource holding potential, genetic quality, and/or reproductive potential [1–3]. Consequently, mate choice decisions are usually made in a non-random way. The pattern of non-random mating, called assortative mating, can be measured as a correlation between male and female phenotypes or genotypes across mated pairs. This correlation can be positive or negative (a.k.a. disassortative) depending on whether individuals select mates based on phenotypic similarity or dissimilarity to themselves, respectively [4]. Nonetheless, assortative mating in humans is most often positive [3].

We should bear in mind, however, that mate choice decisions are not always based on mating preferences. As everybody prefers to have a partner with high resource holding potential, genetic quality, and/or reproductive potential, we may assume that sexual selection acts shaping assortative mating in such a way that women and men exhibiting phenotypic traits associated with high-quality values have advantage over women and men displaying inferior quality values in selecting high-quality partners. Thus, women and men displaying lower quality values have to settle for “choosing” partners of quality similar to themselves [5]. Not surprisingly, couples seeking assisted reproductive technology (ART) treatment exhibit correlations for a range of physical, social, and behavioral characteristics, including age, height, alcohol consumption, education level, smoking, family history of cardiometabolic disease, and ethnicity, as well as for a range of lipids and some other metabolic measures [2]. Note that these correlations involve general traits of human beings. Nevertheless, if we aim to characterize the population of infertile couples, we should focus on traits typically exhibited by individuals suffering from particular diagnoses of infertility. In fact, the scarce literature on this topic shows that infertility diagnoses are genetically and clinically linked with other diseases in single meta-diseases [6]. In addition, infertility diagnoses are associated with non-morbid reproductive and physical/cognitive traits. For example, endometriosis is associated with severe teenage acne, leanness, lower body mass index, high sensitivity to sun exposure, and pigmentary traits such as natural red hair color, light eyes, nevi and freckles. Likewise, semen quality is positively correlated with general intelligence and facial attractiveness [6].

In any case, once a mate “choice” decision has been made, couples that fail to reach a live birth in natural and/or intrauterine insemination (IUI) cycles will likely visit fertility clinics seeking ART treatment. Notably, during the more or less prolonged period of infertility experienced by these couples, those couples with mild/moderate reproductive anomalies would have advantage over couples displaying more severe reproductive alterations in achieving a natural or IUI conception. Thus, we can expect to find a progressive increase in the proportion of couples with more severe reproductive anomalies as duration of infertility rises. In this study, we aim to ascertain whether there is an association between male and female infertility diagnoses (exposures) and duration of infertility (outcome) in couples seeking ART treatment for the first time.

## Material and methods

### Study design

This is a cross-sectional analysis of 1383 infertile couples that sought ART treatment for the first time. These couples entered into our ART program from January 2009 to November 2018 [7]. Female infertility evaluation was performed in accordance with the guidelines laid down by the Spanish Fertility Society (SEF) and the European Society of Human Reproduction and Embryology (ESHRE). Types of semen alterations were classified following the nomenclature and definitions described in the latest edition of the World Health Organization (WHO) manual available at the time couples sought ART treatment. Specifically, the fourth edition [8] from January 2009 until June 2010, and the fifth edition [9] from July 2010 onwards. Couples with a diagnosis of female infertility categorized as “other factors” (n = 33) or whose male partner displayed teratozoospermia (n = 9) or azoospermia (n = 22) were excluded from the study because their sample sizes were limited. Couples that experienced donor IUI cycles or surgical sterilization were also excluded from the study.

### Statistical analysis

Two-sided Pearson’s chi-squared test was applied to test categorical variables and perform a cross-tabulation analysis between “diagnosis of female infertility” and “type of semen alteration”. Likelihood-ratio chi-square statistic was used to estimate asymptotic significances. Forward-stepwise binary logistic regression analyses were applied to calculate exponentiated regression coefficients of independent covariates. Note that, in logistic regression, exponentiated regression coefficients should be interpreted as odds ratios (ORs). Before applying logistic regression analyses, categorical variables were broken down into binary dummy covariates, one for each level of the original categorical variable. Analysis of variance (ANOVA) was applied to compare quantitative data between groups. Values shown in the text and tables are absolute frequencies and percentages, raw/uncorrected means with their 95% confidence intervals (CIs), and exponentiated regression coefficients with their 95% CIs. All the analyses were carried out using the International Business Machines Statistical Package for Social Sciences [IBM SPSS Statistics, version: 28.0.1.1. (14); © Copyright IBM Corporation and its licensors 1989, 2021].

## Results and discussion

Table 1 shows the distribution of “diagnosis of female infertility” and “type of semen alteration” in infertile couples seeking ART treatment for the first time stratified according to whether duration of infertility was ≤ 2 years or > 2 years. No significant differences in the distribution of “diagnosis of female infertility” and “type of semen alteration” between couples exhibiting a duration of infertility ≤ 2 years or > 2 years were evidenced. Note that, despite the median of “duration of infertility” (2.0 years) was chosen as the cut-off point to establish the two groups of duration of infertility, 55.1% and 44.9% of couples were allocated to the ≤ 2 years and > 2 years group, respectively. These percentages deviated from the expected value of 50% because the distribution of the original data displayed a slight positive skewness. That is, more values of “duration of infertility” were on the left side of the distribution, whereas the tail of the distribution was longer on the right side (minimum and maximum values: 0 and 14 years, respectively). Consequently, the mean of the data was greater (2.7 years) than the median (2.0 years) and the mode (2.0 years).

**Table 1:** Distribution of “diagnosis of female infertility” and “type of semen alteration” in infertile couples seeking ART treatment for the first time stratified by “duration of infertility”

	Duration of infertility	
	≤ 2 years	>2 years
Diagnosis of female infertility		
Tubal factor	56.4 (53/94)^a^	43.6 (41/94)
Uterine factor	51.7 (92/178)	48.3 (86/178)
Endometriosis	50.6 (39/77)	49.4 (38/77)
Ovulatory dysfunction	58.0 (80/138)	42.0 (58/138)
Diminished ovarian reserve in women < 35 years	57.1 (36/63)	42.9 (27/63)
Diminished ovarian reserve in women ≥ 35 years	51.2 (41/80)	48.8 (39/80)
Idiopathic	56.1 (202/360)	43.9 (158/360)
Multiple female factors	55.7 (219/393)	44.3 (174/393)
Total	55.1 (762/1383)	44.9 (621/1383)
Type of semen alteration		
Oligozoospermia	53.1 (78/147)	46.9 (69/147)
Asthenozoospermia	58.4 (178/305)	41.6 (127/305)
ACOAT	49.3 (137/278)	50.7 (141/278)
Oligoasthenozoospermia	50.0 (63/126)	50.0 (63/126)
Oligoteratozoospermia	66.7 (2/3)	33.3 (1/3)
Asthenoteratozoospermia	5.0 (14/43)	10.4 (29/43)
Oligoasthenoteratozoospermia	20.9 (58/106)	17.3 (48/106)
Idiopathic	56.5 (369/653)	43.5 (284/653)
Total	55.1 (762/1383)	44.9 (621/1383)

*ACOAT* any combination of oligo-, astheno-, and teratozoospermia; *ART* assisted reproductive technology

^a^Values are percentages and counts in parenthesis

Table 2 shows the distribution of infertile couples seeking ART treatment for the first time according to their diagnoses of female infertility and types of semen alteration, and whether they exhibited a duration of infertility ≤ 2 or > 2 years. As this distribution was not homogeneous (P ≤ 0.001), we applied a forward-stepwise logistic regression analysis in order to ascertain which specific diagnoses of female infertility and/or types of semen alteration were associated with couples’ odds of having a duration of infertility > 2 years. After converting all the categories of “diagnosis of female infertility” and “type of semen alteration” into binary dummy variables, only the covariate “any combination of oligo-, astheno-, and teratozoospermia” (ACOAT) entered into the logistic regression model [OR (95% CI): 1.340 (1.030–1.744)]. That is, being a man suffering from ACOAT was associated with increased couples’ odds of having a duration of infertility > 2 years by a multiplicative factor of 1.340 compared with men that did not exhibit ACOAT. Not surprisingly, the percentage of ACOAT men evidenced in the > 2 years group was significantly higher than the percentage of ACOAT men found in the ≤ 2 years group (22.7% vs 18.0%, respectively; P ≤ 0.029).

**Table 2:** Cross tabulation^a^ of “diagnosis of female infertility” and “type of semen alteration” in infertile couples seeking ART treatment for the first time stratified by “duration of infertility”

Duration of infertility	Diagnosis of female infertility	Type of semen alteration			
		Oligozoospermia	Asthenozoospermia	ACOAT^b^	Idiopathic
≤ 2 years	Tubal factor	1.9 (1/53)^c^	28.3 (15/53)	9.4 (5/53)	60.4 (32/53)
	Uterine factor	8.7 (8/92)	20.7 (19/92)	16.3 (15/92)	54.3 (50/92)
	Endometriosis	10.3 (4/39)	35.9 (14/39)	10.3 (4/39)	43.6 (17/39)
	Ovulatory dysfunction	13.8 (11/80)	17.5 (14/80)	22.5 (18/80)	46.3 (37/80)
	Diminished ovarian reserve in women < 35 years	16.7 (6/36)	13.9 (5/36)	25.0 (9/36)	44.4 (16/36)
	Diminished ovarian reserve in women ≥ 35 years	19.5 (8/41)	29.3 (12/41)	14.6 (6/41)	36.6 (15/41)
	Idiopathic	9.4 (19/202)	22.3 (45/202)	24.3 (49/202)	44.1 (89/202)
	Multiple female factors	9.6 (21/219)	24.7 (54/219)	14.2 (31/219)	51.6 (113/219)
	Total	10.2 (78/762)	23.4 (178/762)	**18.0 (137/762)**	48.4 (369/762)
> 2 years	Tubal factor	7.3 (3/41)	17.1 (7/41)	22.0 (9/41)	53.7 (22/41)
	Uterine factor	14.0 (12/86)	16.3 (14/86)	30.2 (26/86)	39.5 (34/86)
	Endometriosis	5.3 (2/38)	18.4 (7/38)	34.2 (13/38)	42.1 (16/38)
	Ovulatory dysfunction	15.5 (9/58)	17.2 (10/58)	19.0 (11/58)	48.3 (28/58)
	Diminished ovarian reserve in women < 35 years	18.5 (5/27)	3.7 (1/27)	33.3 (9/27)	44.4 (12/27)
	Diminished ovarian reserve in women ≥ 35 years	5.1 (2/39)	28.2 (11/39)	23.1 (9/39)	43.6 (17/39)
	Idiopathic	14.6 (23/158)	22.8 (36/158)	25.3 (40/158)	37.3 (59/158)
	Multiple female factors	7.5 (13/174)	23.6 (41/174)	13.8 (24/174)	55.2 (96/174)
	Total	11.1 (69/621)	20.5 (127/621)	**22.7 (141/621)**^**d**^	45.7 (284/621)

*ACOAT* any combination of oligo-, astheno-, and teratozoospermia, *ART* assisted reproductive technology

^a^Likelihood-ratio chi-square asymptotic significance (2-sided): P ≤ 0.001

^b^Higher odds of having a duration of infertility > 2 years compared with men that did not exhibit ACOAT [OR (95% CI): 1.340 (1.030–1.744)]

^c^Values are percentages and counts in parenthesis

^d^Value significantly different from ACOAT couples exhibiting a duration of infertility ≤ 2 years (P ≤ 0.029)

In order to find out some clues as to why ACOAT couples had higher odds of having a duration of infertility > 2 years, we compared baseline characteristics of ACOAT couples exhibiting a duration of infertility > 2 years with baseline characteristics of ACOAT couples displaying ≤ 2 years of infertility and non-ACOAT couples with > 2 years of infertility (Table 3). Women paired with ACOAT men from the > 2 years group exhibited shorter menstrual cycles (P ≤ 0.047) and decreased antral follicular count (AFC) values (P ≤ 0.008) and anti-Müllerian hormone (AMH) levels (P ≤ 0.007), underwent IUI treatment less frequently (P ≤ 0.0005), and experienced fewer IUI cycles if they went through IUI treatment (P ≤ 0.028) compared with women paired with non-ACOAT men from the > 2 years group. AFC values (P ≤ 0.013) and serum AMH levels (P ≤ 0.001) were also decreased when compared with women paired with ACOAT men from the ≤ 2 years group. Note that women from the three groups analyzed were of similar age, which indicates that differences among groups in “women’s age” cannot explain the lower AFC and AMH values found in women paired with ACOAT men from the > 2 years. On the other hand, the percentage of smoker men was significantly (P ≤ 0.025) higher in ACOAT couples exhibiting a duration of infertility > 2 years than in ACOAT couples from the ≤ 2 years group. Furthermore, ACOAT men suffering from asthenoteratozoospermia displayed higher odds of having a duration of infertility > 2 years compared with ACOAT men that did not exhibit asthenoteratozoospermia [OR (95% CI): 2.275 (1.144–4.523)].

**Table 3:** Baseline characteristics of ACOAT and non-ACOAT couples seeking ART treatment for the first time stratified by “duration of infertility”

Baseline characteristics	ACOAT couples		Non-ACOAT couples
	Duration of infertility		
	≤ 2 years	>2 years	>2 years
Women’s age (years)	34.7 (34.2–35.3)^a^ n = 137	34.8 (34.3–35.4) n = 141	34.9 (34.6–35.1) n = 480
Men’s age (years)	37.0 (36.2–37.9) n = 137	37.6 (36.8–38.4) n = 141	37.5 (37.0–37.9) n = 480
Women’s BMI (kg/m^2^)	23.0 (22.5–23.7) n = 137	23.9 (23.3–24.6) n = 141	23.6 (23.3–24.0) n = 480
No. of women’s brothers and sisters	1.2 (0.9–1.5) n = 30	1.3 (0.8–1.8) n = 36	1.4 (1.1–1.6) n = 148
No. of men’s brothers and sisters	0.9 (0.6–1.3) n = 33	1.1 (0.8–1.4) n = 41	1.5 (1.3–1.7) n = 167
Female tobacco smoking^b^			
0	75.2 (103/137)^c^	71.6 (101/141)	74.4 (357/480)
≥1	24.8 (34/137)	28.4 (40/141)	25.6 (123/480)
Female tobacco smoking ≥ 1^b^	14.0 (12.0–15.9) n = 34	**15.3 (13.6–16.9)**^**d**^ n = 40	13.4 (12.5–14.2) n = 123
Male tobacco smoking^b^			
0	73.0 (100/137)	59.6 (84/141)	65.8 (316/480)
≥1	27.0 (37/137)	**40.4 (57/141)**^**e**^	34.2 (164/480)
Male tobacco smoking ≥ 1^b^	15.8 (14.1–17.5) n = 37	17.4 (16.1–18.6) n = 57	16.7 (16.0–17.5) n = 164
Length of the menstrual cycle (days)	30.6 (28.6–32.6) n = 137	**29.2 (28.1–30.3)**^**f**^ n = 141	31.5 (30.3–32.8) n = 480
No. of previous IUI cycles			
0	79.6 (109/137)	79.4 (112/141)	47.7 (229/480)
≥1	20.4 (28/137)	**20.6 (29/141)**^**g**^	52.3 (251/480)
No. of previous IUI cycles ≥ 1	1.9 (1.6–2.3) n = 28	**2.1 (1.7–2.6)**^**h**^ n = 29	2.6 (2.5–2.8) n = 251
Women’s medical condition^i^			
Healthy	73.0 (100/137)	73.8 (104/141)	74.6 (358/480)
Diseased	27.0 (37/137)	26.2 (37/141)	25.4 (122/480)
Men’s medical condition^i^			
Healthy	83.2 (114/137)	81.6 (115/141)	87.1 (418/480)
Diseased	16.8 (23/137)	18.4 (26/141)	12.9 (62/480)
Type of ACOAT alteration			**–**
Oligoasthenozoospermia	46.0 (63/137)	44.7 (63/141)	–
Oligoteratozoospermia	1.5 (2/137)	0.7 (1/141)	–
Asthenoteratozoospermia	10.2 (14/137)	**20.6 (29/141)**^**j**^	**–**
Oligoasthenoteratozoospermia	42.3 (58/137)	34.0 (48/141)	–
AFC^k^	16.3 (14.8–17.9) n = 137	**13.9 (12.8–15.0)**^**l,m**^ n = 141	16.2 (15.3–17.0) n = 480
AMH (ng/ml)^k^	2.4 (2.2–2.7) n = 137	**2.0 (1.8–2.1)**^**n,o**^ n = 141	2.4 (2.2–2.6) n = 480
FSH (mIU/ml)^k^	7.0 (6.6–7.5) n = 137	6.9 (6.6–7.3) n = 141	7.1 (6.7–7.6) n = 480
LH (mIU/ml)^k^	6.4 (6.0–6.9) n = 137	8.6 (4.3–13.0) n = 141	6.8 (6.2–7.4) n = 480
E_2_ (pg/ml)^k^	54.1 (47.9–60.3) n = 137	51.9 (47.5–56.4) n = 141	53.5 (50.1–56.8) n = 480
TSH (µIU/ml)^k^	3.1 (1.1–5.0) n = 137	2.1 (2.0–2.3) n = 141	2.2 (2.1–2.3) n = 480
PRL (ng/ml)^k^	23.5 (21.0–26.1) n = 137	23.8 (21.5–26.1) n = 141	23.9 (19.5–28.4) n = 480

*ACOAT* any combination of oligo-, astheno-, and teratozoospermia, *AFC* antral follicular count, *AMH* anti-Müllerian Hormone, *ART* assisted reproductive technology, *BMI* body mass index, *CI* confidence interval, *E*_*2*_ estradiol, *FSH* follicle-stimulating hormone, *IUI* intrauterine insemination, *LH* luteinizing hormone, *PRL* prolactin, *TSH* thyroid stimulating hormone

^a^Values are means and 95% CI in parenthesis

^b^Number of cigarettes smoked per day for the 3 months before seeking ART treatment

^c^Values are percentages and counts in parenthesis

^d,f^^,g,h,m,o^Value or distribution significantly different from non-ACOAT couples exhibiting a duration of infertility > 2 years (^d^P ≤ 0.037; ^f^P ≤ 0.047; ^g^P ≤ 0.0005; ^h^P ≤ 0.028; ^m^P ≤ 0.008; ^o^P ≤ 0.007)

^e,l^^,n^Value or distribution significantly different from couples exhibiting a duration of infertility ≤ 2 years (^e^P ≤ 0.018; ^l^P ≤ 0.013; ^n^P ≤ 0.001)

^i^Heathy patients did not report any chronic or acute disease. Diseased patients reported at least one chronic or acute disease. Diseases were assessed following the International Statistical Classification of Diseases and Related Health Problems 10th Revision (ICD-10, Version: 2016) [10]

^j^Higher odds of having a duration of infertility > 2 years compared with ACOAT men that did not exhibit asthenoteratozoospermia [OR (95% CI): 2.275 (1.144–4.523)]

^k^Values on the 3rd day of an unstimulated menstrual cycle

Next, we focused our attention on ascertaining whether the higher percentage of smoker men evidenced in ACOAT couples displaying a duration of infertility > 2 years was associated with specific categories of “type of ACOAT alteration”. In order to reach this aim, we applied a forward-stepwise logistic regression analysis entering the different types of ACOAT alterations as independent covariates. The analysis was exclusively focused on the subpopulation of smoker ACOAT men. Only the covariate “asthenoteratozoospermia” was significant [OR (95% CI): 4.048 (1.082–15.144)]. Specifically, being a smoker and asthenoteratozoospermic man was associated with increased odds of having a duration of infertility > 2 years by a multiplicative factor of 4.048 compared with smoker men that did not exhibit asthenoteratozoospermia. Then, we tested whether being a nonsmoker and asthenoteratozoospermic man was also associated with couples’ odds of having a duration of infertility > 2 years. This analysis showed that being a nonsmorker and asthenoteratozoospermic man (n = 25) was not significantly associated with couples’ odds of having a duration of infertility > 2 years compared with nonsmoker and non-asthenoteratozospermic men (n = 159), [OR (95% CI): 1.618 (0.692–3.784)]. Collectively, these data indicated that asthenoteratozoospermia was not itself directly associated with odds of having a duration of infertility > 2 years. Its association with an extended duration of infertility was an indirect effect induced by male smoking. This inference is endorsed by a previous study [11] showing that smoking is significantly associated with higher incidence of asthenoteratozoospermia in men with primary infertility.

In addition, Table 3 shows that smoker women from the > 2 years group paired with ACOAT men smoked a significantly (P ≤ 0.037) higher number of cigarettes per day than smoker women paired with non-ACOAT men. Differences in number of cigarettes smoked by women from ACOAT and non-ACOAT couples among the distinct categories of “diagnosis of female infertility” were non-significant (P ≤ 0.961). Taken together, these data revealed that a relative low but significant percentage of ACOAT men suffering from asthenoteratozoospermia [10.6% (15/141)] and smoker women paired with ACOAT men [28.4% (40/141)] from the > 2 years group were characterized by their smoking habits. It has been reported that smoking may have negative effects on female [12] and male [13] fertility, although these relationships are likely weak [14]. Furthermore, infertility, especially among women, is associated with psychological distress, impaired couple’s sexual function and social relationships with family and friends, and depressive and anxiety behavior [12, 15, 16]. It is important to remark this point because there is evidence suggesting that smokers have lower distress tolerance (defined as the ability to withstand uncomfortable states) [17]; and stress biomarkers in women from couples attempting to conceive via timed intercourse are associated with longer time to conception and lower chances of conceiving [16]. Thus, the psychological distress that many infertile couples experience during the more or less prolonged duration of infertility may be directly related with higher couples’ smoking habits, longer time to conception, and decreased probabilities of conceiving. Unfortunately, we have no information about the psychological and emotional state of our population of infertile patients and cannot discriminate between the independent effects of smoking and infertility-associated psychological distress on duration of infertility.

Note that despite AFC values and levels of serum AMH were decreased in women paired with ACOAT men from the > 2 years group, only a relative low percentage of women presented values of these biomarkers below the cut-off points adopted by the ESHRE to define poor ovarian responders in ART cycles [18]. In particular, 9.2% (13/141) of women had < 7 antral follicles, and 22.7% (32/141) of women displayed AMH levels < 1.3 ng/ml. That is, the majority of women from ACOAT couples exhibiting a duration of infertility > 2 years cannot be classified as poor ovarian responders following the ESHRE criteria. Hence, we focused our attention exclusively on women that exhibited values of AFC and AMH above the ESHRE cut-off points. This analysis showed that women from ACOAT couples with > 2 years of infertility presented significantly decreased AFC and AMH values when compared with women from ACOAT couples with ≤ 2 years of infertility and women from non-ACOAT couples with > 2 years of infertility (Table 4). That is, our data indicate that the general subpopulation of women paired with ACOAT men from the > 2 years group was characterized by having decreased ovarian reserve. The shorter menstrual cycle length displayed by these women compared with women from non-ACOAT couples (Table 3) endorses this inference. In fact, shorter menstrual cycles within the normal range are associated with significant decreases in AFC values and serum AMH levels, the most reliable biomarkers of ovarian reserve considered today [19].

**Table 4:** AFC and AMH values above the ESHRE cut-off points in women from ACOAT and non-ACOAT couples seeking ART treatment for the first time stratified by “duration of infertility”

Baseline characteristics	ACOAT couples		Non-ACOAT couples
	Duration of infertility		
	≤ 2 years	>2 years	>2 years
AFC^a^	16.9 (15.3–18.5)^b^ n = 130	**14.8 (13.6–15.9)**^**c,d**^ n = 128	17.2 (16.4–18.1) n = 437
AMH (ng/ml)^a^	2.8 (2.6–3.0) n = 113	**2.3 (2.2–2.4)**^**e,f**^ n = 109	2.8 (2.7–3.0) n = 377

*ACOAT* any combination of oligo-, astheno-, and teratozoospermia, *AFC* antral follicular count, *AMH* anti-Müllerian Hormone, *ART* assisted reproductive technology, *ESHRE* European Society of Human Reproduction and Embryology

^a^Values on the 3rd day of an unstimulated menstrual cycle

^b^Values are means and 95% CI in parenthesis

^c,e^Value significantly different from ACOAT couples exhibiting a duration of infertility ≤ 2 years (^c^P ≤ 0.029; ^e^P ≤ 0.001)

^d,f^Value significantly different from non-ACOAT couples exhibiting a duration of infertility > 2 years (^d^P ≤ 0.005; ^f^P ≤ 0.002)

Interestingly, shorter menstrual cycles and reduced AFC values and serum AMH levels are closely associated with lower odds of pregnancy (fecundability) in natural cycles as well as clinical pregnancy after ART treatment [19]. Our data are in line with these findings. Actually, the odds of clinical pregnancy and live birth in the first autologous fresh IVF/ICSI cycle displayed by women from ACOAT couples with a duration of infertility > 2 years were significantly lower than the odds exhibited by women from ACOAT couples from the ≤ 2 years group [OR (95% CI): 0.524 (0.303–0.906) for clinical pregnancy and 0.415 (0.219–0.786) for live birth], and women from non-ACOAT couples from the > 2 years group [OR (95% CI): 0.584 (0.369–0.923) for clinical pregnancy and 0.407 (0.235–0.703) for live birth].

We may assume that couples consisting of ACOAT men and women with a relative low ovarian reserve progressively accumulate as duration of infertility extends because men exhibiting normozoospermia or mild/moderate semen alterations and women with larger ovarian reserve have higher probabilities of natural and IUI conception after they make the decision to have a child. Two lines of evidence provide indirect clues pointing out that ACOAT men indeed had more severely impaired semen quality compared with the other categories of semen alterations included in the present study. Firstly, Table 3 shows that ACOAT couples underwent fewer previous IUI cycles than non-ACOAT couples. And secondly, the percentage of couples allocated to ICSI treatment when entered into our ART program was similar in ACOAT couples from the > 2 years group (67.4%, 95/141) and the ≤ 2 years group (69.3%, 95/137). In contrast, only 16.7% (80/480) of non-ACOAT couples with a duration of infertility > 2 years were assigned to ICSI treatment.

## Strengths and limitations

One strength of the present study lies in the fact that it is exclusively focused on a population of infertile couples that sought ART treatment for the first time. The cross-sectional design of the study allowed us to have a precise reference point to perform comparisons between groups and, even more importantly, it has enabled us to propose for the first time a hypothesis on the origin of couples’ infertility (see the Introduction section). Furthermore, all the data analyzed came from a single center. This methodology has the advantage of reducing heterogeneity among couples due to genetic and environmental factors. However, the generalizability of the results for other populations of infertile couples may be restricted. Another limitation of the present study stems from the fact that data on duration of infertility, number of previous IUI cycles, and couples’ medical condition were based on interviews at the first visit to our ART Unit. Thus, these data rely on the subjective information given by the patient. Finally, types of semen alterations were categorized following the nomenclature and definitions described by WHO manual available at the time couples sought ART treatment. Detailed data on particular semen parameters were not available on our database. The inclusion and analysis of detailed semen parameters would have improved the categorization of men according to the severity degree of semen alterations.

## Conclusions

Overall, this study shows that couples consisting of ACOAT men and women with a relative low ovarian reserve are overrepresented in couples seeking ART treatment for the first time after experiencing > 2 years of infertility. This outcome has led us to develop a general hypothesis proposing that the origin of couple’s infertility is a consequence of a process of positive assortative mating shaped by sexual selection for physical and cognitive traits. Further work is needed to untangle the phenotypic relationships that may be present between different female and male infertility diagnoses. This information will allow us to ascertain whether women suffering from a particular diagnosis of infertility tend to pair with men displaying a specific type of semen alteration and vice versa.

## Data Availability

The datasets used and/or analyzed during the current study are available from the corresponding author on reasonable request.

## References

[1] Huber S, Fieder M (2011). Educational homogamy lowers the odds of reproductive failure. PLoS ONE.

[2] Al Rashid K, Goulding N, Taylor A, Lumsden MA, Lawlor DA, Nelson SM (2021). Spousal associations of serum metabolomic profiles by nuclear magnetic resonance spectroscopy. Sci Rep.

[3] Pisanski K, Fernandez-Alonso M, Díaz-Simón N, Oleszkiewicz A, Sardinas A, Pellegrino R (2022). Assortative mate preferences for height across short-term and long-term relationship contexts in a cross-cultural sample. Front Psychol.

[4] Jiang Y, Bolnick DI, Kirkpatrick M (2013). Assortative mating in animals. Am Nat.

[5] Prall S, Scelza B (2022). The effect of mating market dynamics on partner preference and relationship quality among *Himba** pastoralists*. Sci Adv.

[6] Tarín JJ, García-Pérez MA, Hamatani T, Cano A (2015). Infertility etiologies are genetically and clinically linked with other diseases in single meta-diseases. Reprod Biol Endocrinol.

[7] Tarín JJ, Pascual E, Gómez R, García-Pérez MA, Cano A (2020). Predictors of live birth in women with a history of biochemical pregnancies after assisted reproduction treatment. Eur J Obstet Gynecol Reprod Biol.

[8] WHO (1999). Laboratory manual for the examination of human semen and sperm-cervical mucus interaction.

[9] WHO (2010). Laboratory manual for the examination and processing of human semen.

[10] Tarín JJ, Pascual E, García-Pérez MÁ, Gómez R, Cano A (2019). Women’s morbid conditions are associated with decreased odds of live birth in the first IVF/ICSI treatment: a retrospective single-center study. J Assist Reprod Genet.

[11] Gaur DS, Talekar M, Pathak VP (2007). Effect of cigarette smoking on semen quality of infertile men. Singapore Med J.

[12] Nik Hazlina NH, Norhayati MN, Shaiful Bahari I, Nik Muhammad Arif NA (2022). Worldwide prevalence, risk factors and psychological impact of infertility among women: a systematic review and meta-analysis. BMJ Open.

[13] Durairajanayagam D (2018). Lifestyle causes of male infertility. Arab J Urol.

[14] Hernáez Á, Wootton RE, Page CM, Skåra KH, Fraser A, Rogne T (2022). Smoking and infertility: multivariable regression and Mendelian randomization analyses in the Norwegian Mother, Father and Child Cohort Study. Fertil Steril.

[15] Cocchiaro T, Meneghini C, Dal Lago A, Fabiani C, Amodei M, Miriello D (2020). Assessment of sexual and emotional distress in infertile couple: validation of a new specific psychometric tool. J Endocrinol Invest.

[16] Dube L, Bright K, Hayden KA, Gordon JL (2022). Efficacy of psychological interventions for mental health and pregnancy rates among individuals with infertility: a systematic review and meta-analysis. Hum Reprod Update..

[17] Veilleux JC (2019). The relationship between distress tolerance and cigarette smoking: a systematic review and synthesis. Clin Psychol Rev.

[18] Ferraretti AP, Gianaroli L (2014). The Bologna criteria for the definition of poor ovarian responders: is there a need for revision?. Hum Reprod.

[19] Younis JS, Iskander R, Fauser BCJM (2020). Izhaki I 2020 Does an association exist between menstrual cycle length within the normal range and ovarian reserve biomarkers during the reproductive years? A systematic review and meta-analysis. Hum Reprod Update.

